# Notes and News

**Published:** 1897-11-13

**Authors:** 


### Notes and News.

(Collected and Communicated.)

So great has been the activity in hospital building during this Jubilee year, that our columns are in danger of being flooded with records of new structures. It is a flood we would not wish to stem, and the result is likely to mark an epoch in history, as showing the greatest efforts ever made to alleviate the suffering poor.

It is reported that yellow fever is gaining ground in Jamaica, and that amongst its victims are several military and naval officers.

Mr. Passmore Edwards has laid the foundation stone of a Convalescent Home at Herne Bay, which he is presenting to the members of the Friendly Societies. The building is to accommodate 50 persons, and it is to cost £6,000.

Dr. Alfredo A. Kanthack, F.R.C.S., Lecturer on Pathology at St. Bartholomew's Hospital, has been elected to the professorship in that subject at Cambridge University, in succession to the late Dr. C. S. Roy. Dr. Kanthack was a member of the Indian Leprosy Commission.

Sir William Broadbent, Bart., M.D., F.R.S., will preside at Sir Squire Bancroft's reading of Dickens's "Christmas Carol" at Portman Rooms on November 22nd, in aid of St. Mary's Hospital. This is the first of the series which Sir Squire Bancroft will give in London during the coming winter.

Typhoid fever has gained a very serious hold upon Lynn, and a large number of deaths have occurred. At Maidstone fresh cases continue to occur, though not in large numbers, and the number of persons attacked exceeds 1,830. The Prince of Wales has telegraphed his heartfelt sympathy with the afflicted town.

Through the generosity of Mr. John Davies, of Wyeside, Builth has been provided with a cottage hospital. The hospital, which faces south and is situated on a beautiful site, contains two general and two accident wards and day rooms for the patients, the usual offices, and accommodation for the staff. A verandah runs in front of the building, and is continued for a short distance along the sides. The executors of Mr. Davies will set aside £6,000 for the purpose of establishing the hospital, £2,700 of which goes for site, buildings, and furnishing, the remainder being put to the Endowment Fund.

Lady Mary Howard has opened the new buildings at the Royal Infirmary, Sheffield. These consist of eye wards, laundry, and nurses' home. The latter is not yet completed.

The sum of £500 provisionally promised to the Devon and Exeter Hospital has been secured. The condition was that £1,500 should be subscribed by others towards freeing the hospital from debt by a certain date. On the day fixed over the required sum had been secured, a fact which reflects much credit on all concerned, considering the short time in which the money was collected.

The Dowager Countess of Erroll opened the new block added to the Royal Aberdeen Infirmary in commemoration of the Queen's long reign. The Countess delivered a message from Her Majesty, expressing her appreciation of the good work already achieved by the institution and her good wishes for its future. The infirmary is now complete, and possesses 50 beds.

The proprietors of the *Graphic* and *Daily Graphic* are to be congratulated on the splendid result of their excellent endeavours on behalf of the Prince of Wales's Hospital Fund. They have sent in to the hon. secretaries of the Fund cheques for £3,486, representing a profit of £2,270 on the sale of the illustrated programme of the Jubilee procession, and £1,215 subscribed through the columns of the *Daily Graphic*. Still more valuable is the promise the proprietors have received of £815 in annual subscriptions.

For some time past an impression has crept abroad that the Cardiff Infirmary is not well managed, and an accusation of extravagant expenditure has been brought against it. At the last general meeting of the Infirmary Committee a committee was formed to inquire into the administration. Whether the complaints made are justifiable or not, a rule prohibiting visitors from bringing food or alcoholic liquors into the institution should certainly be made. It is strange that so necessary a rule should not have been made before.

The new Middle Ward Hospital for Lanarkshire, now opened near Motherwell, is the finest and largest county hospital in Scotland. It contains four pavilions of 20 beds each, and eight single wards; one isolation pavilion for six beds, and one observation pavilion containing the same number. At the south end of each pavilion is a verandah. Although the accepted lines of hospital construction of this kind have been followed, the cost of the building amounts to £370 per bed, including all furniture and fittings.

The new general hospital at Kettering is now open. The originator of the scheme to erect a hospital was Miss Mary Stockburn, whose father headed the subscription-list for the purpose with a donation of £500. The new hospital contains operating theatre, private ward, dispensary, and other offices in the central block. In pavilions on each side are situated the wards, one for male and one for female patients, with ten beds and two cots in each. These run east and west, but large bay windows are placed at the south end of each. The cost of the building has been £10,000, nearly all of which is subscribed.

